# Detecting Changes in Tissue Perfusion With Hyperspectral Imaging and Thermal Imaging Following Endovascular Treatment for Peripheral Arterial Disease

**DOI:** 10.1177/15266028221082013

**Published:** 2022-03-08

**Authors:** Simone F. Kleiss, Kirsten F. Ma, Mostafa El Moumni, Çagdas Ünlü, Thomas S. Nijboer, Richte C. L. Schuurmann, Reinoud P. H. Bokkers, Jean-Paul P. M. de Vries

**Affiliations:** 1 Department of Surgery, Division of Vascular Surgery, University Medical Center Groningen, University of Groningen, Groningen, The Netherlands; 2 Department of Surgery, Division of Trauma Surgery, University Medical Center Groningen, University of Groningen, Groningen, The Netherlands; 3 Department of Vascular Surgery, Noordwest Hospital Group, Alkmaar, The Netherlands; 4 Department of Radiology, Medical Imaging Center, University Medical Center Groningen, University of Groningen, Groningen, The Netherlands

**Keywords:** endovascular procedures, hyperspectral imaging, peripheral arterial disease, thermal imaging, tissue perfusion

## Abstract

**Purpose:**

Hyperspectral imaging (HSI) and thermal imaging allow contact-free tissue perfusion measurements and may help determine the effect of endovascular treatment (EVT) in patients with peripheral arterial disease. This study aimed to detect changes in perfusion with HSI and thermal imaging peri-procedurally and determine whether these changes can identify limbs that show clinical improvement after 6 weeks.

**Methods:**

Patients with Rutherford class 2–6 scheduled for EVT were included prospectively. Hyperspectral imaging and thermal imaging were performed directly before and after EVT. Images were taken from the lateral side of the calves and plantar side of the feet. Concentrations of (de)oxyhemoglobin, oxygen saturation, and skin temperature were recorded. Angiographic results were determined on completion angiogram. Clinical improvement 6 weeks after EVT was defined as a decrease ≥ one Rutherford class. Peri-procedural changes in perfusion parameters were compared between limbs with and without good angiographic results or clinical improvement. To identify limbs with clinical improvement, receiver operating characteristic (ROC) curves were used to determine cutoff values for change in HSI.

**Results:**

Included were 23 patients with 29 treated limbs. Change in HSI values and temperature was not significantly different between limbs with good and poor angiographic results. Change in peri-procedural deoxyhemoglobin, determined by HSI, at the calves and feet was significantly different between limbs with and without clinical improvement at 6 week follow-up (p=0.027 and p=0.017, respectively). The ROC curve for change in deoxyhemoglobin at the calves showed a cutoff value of ≤1.0, and ≤−0.5 at the feet, which were discriminative for clinical improvement (sensitivity 77%; specificity 75% and sensitivity 62%; specificity 88%, respectively).

**Conclusions:**

HSI can detect changes in perfusion at the calves after EVT in patients with Rutherford class 2–6. Peri-procedural deoxyhemoglobin changes at the calves and feet are significantly different between limbs with and without clinical improvement. Decrease in deoxyhemoglobin directly after EVT may identify limbs that show clinical improvement 6 weeks after EVT.

## Introduction

Peripheral arterial disease (PAD) affects millions of people worldwide. The prevalence of PAD increases with age to 20% at the age of 80 years.^1^ Endovascular treatment (EVT) is the preferred revascularization method for restoring blood flow to the lower extremity and feet in patients with PAD. To date, technical success of EVT is per procedurally assessed with digital subtraction angiography (DSA).^2^ Even when technically successful target lesion revascularization is observed on DSA, clinical outcomes after revascularization are still rather unpredictable, with substantial rates of minor and major amputations and non-healing ulcers.^3–5^ Other diagnostic techniques with a focus on tissue perfusion measurements may be used to assess the efficacy of revascularization therapy, although evidence is still scarce.^6,7^

Hyperspectral imaging (HSI) and thermal imaging have been developed for non-invasive assessment of tissue perfusion.^8^ The penetration depth of HSI is 1 to 2 mm. The hand-held HSI camera uses visible light spectroscopy to determine concentrations of oxyhemoglobin (OxyHb) and deoxyhemoglobin (DeoxyHb), which are presented in a colour-coded image.^9^ Thermal imaging measures the skin temperature with an infrared camera. Skin temperature is a derivative of the blood flow and, therefore, a potential measure of skin perfusion.^10^

Previous studies found differences in HSI values in patients with PAD compared with controls and a significant correlation with severity of disease. Besides that, HSI and thermal imaging have shown a good test-retest reliability for perfusion measurements.^11–15^ No studies to date have evaluated improvement of tissue perfusion with HSI after EVT. The measurement of temperature changes of the lower extremity after EVT have been investigated; however, the studies demonstrate conflicting results.^16,17^ Immediate assessment of tissue perfusion after EVT with HSI or thermal imaging could be used as an endpoint to determine success of revascularization.

We hypothesize that HSI and thermal imaging can detect changes in perfusion in the lower extremity directly after EVT. We therefore performed HSI together with thermal imaging directly before and after endovascular revascularization procedures in the lower leg. We aimed to determine whether changes in HSI pre- and post-EVT are different in patients with and without successful intervention and which of the peri-procedural perfusion parameters are able to identify limbs that show clinical improvement 6 weeks after endovascular revascularization.

## Methods

This single-center prospective cohort study enrolled patients undergoing EVT from November 2019 to March 2021. The study protocol was approved by the University Medical Center Groningen (UMCG) Institutional Review Board (IRB #2019/114). Study procedures were performed according to the Medical Research Involving Human Subjects Act and Declaration of Helsinki. Written informed consent was obtained from all patients. Patients were enrolled as part of a large observational study (Netherlands Trial Register #7713) evaluating non-invasive diagnostic methods for monitoring of tissue perfusion in vascular patients.

### Study Patients

Patients with PAD scheduled for EVT at the UMCG were eligible for inclusion in this study. Inclusion criteria were age >18 years, presence of Rutherford classification^18^ 2–6, and a clinical indication for elective endovascular revascularization. Exclusion criteria were severe peripheral oedema, cellulitis, or erysipelas of the treated leg or foot.

Baseline patient demographics including sex, age, body mass index, smoking status, comorbidities, and the Rutherford classification and ankle brachial indices (ABIs) were collected form electronic medical records. A multidisciplinary team of vascular surgeons and interventional radiologists determined the indication for EVT based on clinical evaluation, Doppler ultrasound imaging, and computed tomography (CT) angiography.

### Endovascular Revascularization

EVT was performed under local anesthesia in an angiography suite. Target lesions were treated with percutaneous transluminal angioplasty (PTA) and with additional stent placement in case of acute recoil, flow-limiting dissections, or >30% residual stenosis measured on angiography.^19^ Technical success of revascularization of the target lesion was defined as <30% residual stenosis present on the completion angiogram examined by the executing interventionalist.^2^ The entire completion angiography run, including images without contrast, were forwarded integrally to the picture archiving and communication system to ensure the dynamic aspect of the angiography was not lost.

All lesions were scored according to the TransAtlantic Inter-Society Consensus for Management of Peripheral Arterial Disease (TASC-II) classification by the executing interventionalist as well.^20^ The likelihood of leading to increased perfusion to the lateral side of the calves and plantar side of the foot were further classified by the two vascular specialists (J.P.V. and R.B.), blinded to the results of the tissue perfusion imaging results. The angiographic result at the lateral side of the calves was considered “good” if a TASC-II C or D lesion was treated and uninterrupted flow was present in the femoral-popliteal arteries continuing into the tibial anterior artery. The angiographic result of the feet was considered “good” if a TASC-II C or D lesion was treated and uninterrupted flow was present in at least one below-the-knee artery with continuation to the infra-malleolar arteries. Completion angiographies (for calves and feet) that did not meet these criteria were classified as “poor” angiographic result, because a change in peripheral perfusion was not expected in these limbs directly after EVT. Angiographic results in which there was disagreement were discussed to reach consensus. Classification of angiographic results by the two experienced vascular specialists was unanimous in 26 (90%) cases and discussion followed to reach consensus in 3 (10%) cases.

### Imaging Procedures

Hyperspectral imaging and thermal imaging were performed in supine position ≤10 minutes before and ≤10 minutes after EVT in the angiography suite. The room temperature was set to 18°C, and patients were in this resting position for at least 15 minutes before to pre-procedural perfusion imaging. Hyperspectral and thermography images were taken from the plantar side of the feet and lateral side of the calves. The measurement location at the lateral side of the calves was marked 5 cm distally from the fibular head. Standardized image acquisition with both cameras was performed perpendicular to the skin at 38 cm, which is in accordance with the instructions for use.

### Hyperspectral Imaging

Hyperspectral imaging was performed with a HyperView camera system (HyperMed Inc, Memphis, TN, USA), which uses wavelengths of visible light to measure the concentration of OxyHb and DeoxyHb in the upper 1 to 2 mm of the skin.^21^ The concentrations are presented in a color-coded image, with each pixel presenting the amount of OxyHb and DeoxyHb. Oxygen saturation is calculated by the HyperMed software using these concentrations with the following formula.

(1) $$\mathrm{Oxygen}\;\mathrm{saturation}\;=\;\mathrm{OxyHb}/(\begin{array}{l} \mathrm{OxyHb}\;+\;\\ \mathrm{DeoxyHb} \end{array})\;\times \;100\%.$$

The hyperspectral images were analyzed using the software provided on the system by HyperMed Inc. A circular 16 mm diameter region of interest (ROI) was placed on the hyperspectral images at the plantar side of the feet, at the caput of the third metatarsus, and at the lateral side of the lower leg on the previously described marked location 5 cm distally from the fibular head.

### Thermal Imaging

**Figure 1.**
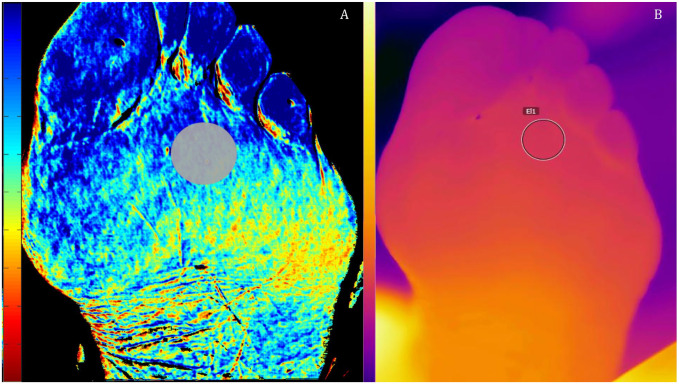
Hyperspectral image of the deoxyhemoglobin concentration (A) and thermal image (B) of the plantar side of the foot of one of the study patients. The circles in both figures mark the region of interest.

Thermal imaging was performed with a FLIR camera T1030sc (FLIR Systems Inc, Wilsonville, OR, USA). Thermal imaging uses an infrared camera to provide a temperature map based on the emissive radiation of an object.^10^ The thermal images were recorded subsequently to the hyperspectral images. For analysis of the thermal images, a similar ROI was placed at the same location as the ROI used for hyperspectral images using FLIR Systems software. A hyperspectral image and a thermal image of a foot of one of the included patients are shown in Figure 1.

### Follow-up

Post-EVT clinical outcomes were obtained from the outpatient clinic visit at the department of vascular surgery 6 weeks after EVT. Duplex ultrasound imaging was performed according to standard of care in order to determine the patency of the revascularization. Clinical improvement after 6 weeks was defined as at least one class improvement in the Rutherford classification.

### Statistical Analysis

Data were collected using REDCap (Vanderbilt University, Nashville, TN, USA), an online data management service. Statistical analyses were performed using SPSS 23 software (IBM Corp, Armonk, NY, USA). According to data distribution, descriptive statistics are presented as median with 25th and 75th percentile. Differences between variables before and after EVT were calculated using a Wilcoxon signed rank test. Differences between patients with and without a good angiographic result and between patients with and without improved clinical outcome at 6 weeks were calculated with a Mann-Whitney *U* test. A spearman correlation test was performed to determine the correlation between the change in HSI values and change in temperature directly after EVT. A correlation coefficient of 0.0–0.1 was considered negligible, 0.1–0.39 was considered weak, 0.40–0.69 was considered moderate, 0.7–0.89 was considered strong, and 0.9–1.0 was considered a very strong correlation.^22^ Sensitivity and specificity were calculated with a cross-tabulation to determine the ability of angiographic results directly after EVT to identify limbs that showed clinical improvement 6 weeks after EVT. Receiver operating characteristic (ROC) curves with corresponding area under the curve (AUC) were calculated to determine the optimal cutoff values for change in perfusion parameters directly after EVT to identify limbs that showed clinical improvement 6 weeks after EVT. Cutoff values with corresponding sensitivity and specificity were determined based on the minimum sum of 1−sensitivity and 1−specificity. For all statistical analyses, a p value of ≤0.05 was considered statistically significant.

## Results

### Patients Characteristics

Patients were screened for eligibility from November 2019 to March 2020. After the lockdown due to the coronavirus disease 2019 (COVID-19) pandemic, screening was continued from August 2020 until March 2021. Among 27 patients with an indication for EVT who were screened for eligibility, 2 patients were excluded with peripheral oedema and 2 patients due to HSI image artifacts or absence of peri-procedural HSI or thermal images. The study included 23 patients with 29 index limbs. Of the 29 index limbs, HSI and thermal images of 25 calves and 29 feet were included. Images of 3 calves were missing, due to limited access caused by sterile draping covering the limbs in the angiography suite before EVT. One hyperspectral image of the calf could not be assessed due to artifacts caused by a tattoo near the ROI. Patient characteristics are presented in Table 1.

**Table 1.** Patient Characteristics.

	(N=23)	
Age (years)	66.0	(58.0, 72.0)
Sex		
Male	16	(70%)
Female	7	(30%)
Body mass index (kg/m^2^)	25.2	(23.5, 28.3)
Smoking		
Current	11	(48%)
Ex-smoker	10	(44%)
Non-smoker	2	(8%)
Diabetes mellitus	12	(52%)
Type I	1	(4%)
Type II	11	(48%)
Hypertension	14	(61%)
Pulmonary disease	8	(35%)
Hyperlipidemia	6	(26%)
Coronary artery disease	12	(52%)
Chronic kidney disease	6	(26%)
Prior cerebral events	6	(26%)

Values are presented as median and interquartile range or count with percentage. Pulmonary diseases include chronic obstructive pulmonary disease, asthma, and emphysema. Coronary artery diseases include myocardial infarction, percutaneous coronary intervention, or coronary artery bypass grafting.

### Revascularization Treatment

The characteristics of the treated limbs and arterial segments are presented in Table 2. Of all treated legs, 52% were classified as Rutherford class 4, 5, or 6. Eight limbs suffered Rutherford class 4, 6 limbs suffered Rutherford class 5, and 1 limb suffered Rutherford class 6. In the 29 target limbs, 48 target lesions were treated. In limbs of patients suffering Rutherford class 2–3, the lesions were most frequently (58%) located in the iliac artery, and most often treated with stents (90%). Limbs of patients suffering Rutherford class 4–6 needed treatment most often for femoral lesions (38%) with plain old balloon angioplasty (48%). Plain old balloon angioplasty was performed in 15 out of 48 lesions (31%). In 31 out of 48 lesions (65%), a stent was placed after PTA (16 bare metal stents, 8 drug eluting stents and 7 covered stents). Technical success, <30% residual stenosis, was achieved in 96% of the target lesion revascularizations. For study purposes, the completion angiograms were classified by two experienced vascular specialists. The angiographic classification was performed after and regardless of the EVT. The completion angiograms were classified as a good angiographic result in 11 calves and as a poor angiographic result in 18 calves. The completion angiograms were classified as a good angiographic result in 9 feet and as a poor angiographic result in 19 feet. In one completion angiogram, no images of the foot were available.

**Table 2.** Characteristics of the Treated Limbs With Target Lesions.

Rutherford classification	Rutherford class 2–3		Rutherford class 4–6	
Limbs	n=14		n=15	
Lesions	n=19		n=29	
Treated arterial segments				
Iliac	11	(58%)	5	(17%)
Femoral	7	(37%)	11	(38%)
Popliteal	-	-	6	(21%)
Crural	1	(5%)	7	(24%)
Target lesions				
CIA	8	(42%)	3	(10%)
EIA	7	(37%)	2	(7%)
SFA	3	(16%)	11	(38%)
Popliteal artery	-	-	6	(21%)
ATA	1	(5%)	4	(14%)
Peroneal artery	-	-	3	(10%)
TASC-II classification				
TASC-II A	7	(37%)	8	(28%)
TASC-II B	4	(21%)	10	(34%)
TASC-II C	1	(5%)	2	(7%)
TASC-II D	7	(37%)	9	(31%)
Endovascular treatment				
PTA	2	(10%)	13	(45%)
PTA and stent	17	(90%)	14	(48%)
Unsuccessful	-	-	2	(7%)
Type of balloon or stent				
BMS	11	(58%)	5	(19%)
DES	1	(5%)	7	(26%)
Covered Stents	5	(26%)	2	(7%)
POBA	2	(11%)	13	(48%)

Abbreviations: ATA, anterior tibial artery; BMS, bare metal stent; CIA, common iliac artery; DES, drug eluting stent; EIA, external iliac artery; POBA, plain old balloon angioplasty; PTA, percutaneous transluminal angioplasty; SFA, superficial femoral artery; TASC-II, TransAtlantic Inter-Society Consensus-II.

### Peri-procedural Perfusion Changes

The HSI values and local skin temperature determined directly before and after EVT are presented in Table 3. After EVT, there were no statically significant changes in perfusion values at the calves and the feet for the entire group of patients compared with the pre-EVT measurements.

**Table 3.** HSI Values and Skin Temperature of the Calves and Feet of the Treated Legs Directly Before and After EVT.

		Pre-EVT	Post-EVT	p value
Calves	N=25			
HSI measurements	Oxyhemoglobin (a.u.)	26.0 (16.0, 31.5)	27.0 (19.0, 41.0)	0.492
	Deoxyhemoglobin (a.u.)	41.0 (33.5, 57.5)	38.0 (29.0, 57.0)	0.368
	Oxygen saturation (%)	39.0 (23.5, 49.0)	42.0 (31.0, 52.0)	0.212
	N=26			
Thermal imaging	Temperature (°C)	32.8 (32.2, 34.2)	33.3 (32.2, 34.7)	0.367
Feet	N=29			
HSI measurements	Oxyhemoglobin (a.u.)	78.0 (64.0, 93.5)	76.0 (61.0, 95.0)	0.745
	Deoxyhemoglobin (a.u.)	57.0 (42.5, 81.0)	58.0 (46.5, 73.5)	0.550
	Oxygen saturation (%)	59.0 (48.5, 64.0)	59.0 (48.5, 66.0)	0.427
Thermal imaging	Temperature (°C)	29.6 (25.1, 32.4)	31.3 (25.2, 33.8)	0.247

Values are presented as median and interquartile range. Wilcoxon signed rank test was performed to calculate differences between variables before and after EVT. Included numbers were lower at the calves because of missing images.

Abbreviations: a.u., arbitrary units; EVT, endovascular treatment; HSI, hyperspectral imaging.

**Figure 2.**
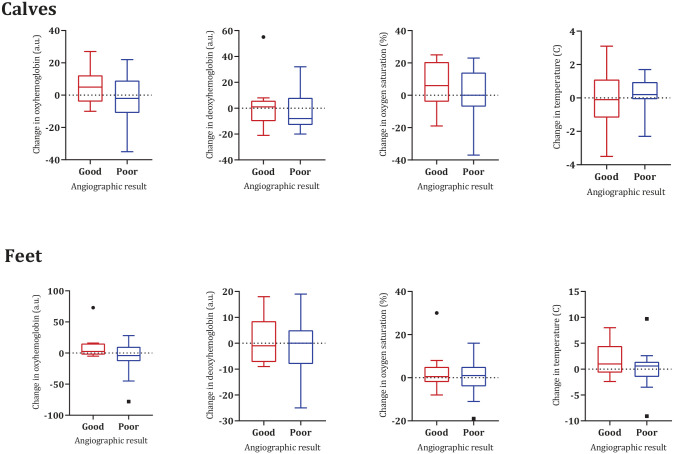
Changes in hyperspectral imaging values and skin temperature at the calves and feet after EVT for limbs with good vs poor angiographic result. Calves: good result HSI n=10, thermal n=11, and poor result n=15. Feet: good result n=9 and poor result n=19. There were no statistically significant differences between the groups. EVT, endovascular treatment; HSI, hyperspectral imaging.

Figure 2 illustrates the changes in HSI values and skin temperatures at the calves and feet comparing the limbs and feet with a good versus a poor angiographic result. There were no statistically significant differences in the change of perfusion parameters between the limbs with good angiographic result compared with limbs with a poor angiographic result.

The perfusion values of the calves and the feet in limbs classified as a good angiographic result are presented in Supplementary Table 1. In these limbs, OxyHb (p=0.050) and oxygen saturation (p=0.050) increased significantly after EVT at the calves. No other significant changes were determined regarding HSI or thermal imaging values in the calves or feet.

### Correlation between Change in HSI and Change in Thermal Imaging

The change in HSI values at the feet showed a weak and non-significant correlation with change in temperature at the feet. The change in OxyHb and oxygen saturation at the calves showed a positive and significant correlation with change in temperature (R=0.51, p=0.009 and R=0.52, p=0.008, respectively). Change in DeoxyHb at the calves showed a negative and significant correlation with change in temperature (R=−0.51, p=0.009).

### Perfusion Changes and Clinical Improvement

After 6 weeks, 21 limbs (72%) showed clinical improvement, 7 limbs (24%) showed an improvement of one or two Rutherford classes, and 14 limbs (48%) improved more than two Rutherford classes. One patient with Rutherford class 5 did not improve and one patient with Rutherford class 6 did not improve. All other patients with Rutherford class 5 showed wound healing after 6 weeks. Twenty-eight limbs were evaluated with duplex ultrasound after 6 weeks. The ultrasound showed no restenoses or occlusions of the treated lesions in these patients. One patient underwent amputation within 6 weeks and therefore had no ultrasound follow-up. Of these 21 limbs with clinical improvement, 21 hyperspectral and thermal images of the feet were available and 17 hyperspectral images and 18 thermal images of the calves.

**Figure 3.**
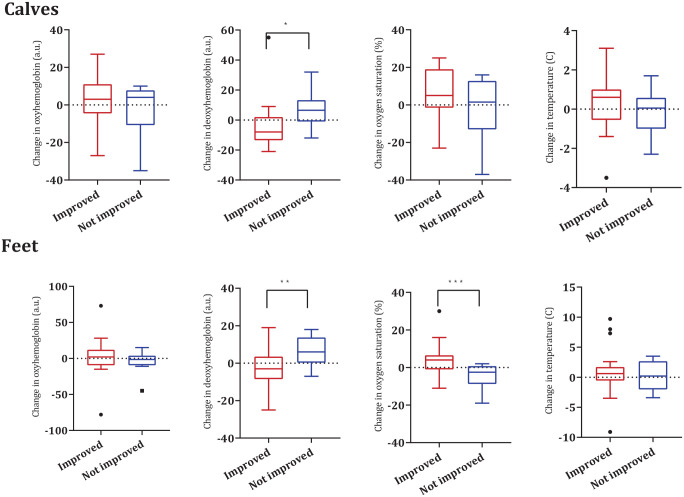
Changes in hyperspectral imaging values and skin temperature at the calves and feet measured directly after EVT in limbs with clinical improvement vs no clinical improvement after 6 weeks. Calves: improved HSI n=17, thermal n=18, and not improved n=8. Feet: improved n=21 and not improved n=8. EVT, endovascular treatment; HSI, hyperspectral imaging. *p=0.027. **p=0.017. ***p=0.014.

Figure 3 presents the changes in HSI values and skin temperature at the feet and calves directly after EVT in limbs that showed clinical improvement vs limbs that did not improve after 6 weeks. Limbs with clinical improvement after 6 weeks had a decrease in DeoxyHb at the calves and feet after EVT, which was significantly different from the increase in DeoxyHb at the calves and feet in limbs without clinical improvement (p=0.027 and p=0.017, respectively). Also limbs with improvement showed an increase in oxygen saturation at the feet which were significantly different from the decrease in oxygen saturation in the limbs without improvement (p=0.014).

The HSI values and local skin temperature determined in these 21 limbs directly before and after EVT are presented in Supplementary Table 2. The levels of DeoxyHb measured at the calves decreased significantly after EVT (p=0.010). The levels of DeoxyHb at the feet decreased substantially, but not significantly after EVT (p=0.056). No other significant changes were determined regarding HSI or thermal imaging values in the calves nor feet.

### Sensitivity and Specificity of Completion Angiographic Result for Clinical Improvement at 6 Weeks

Good angiographic result at the calves had a sensitivity of 38% and a specificity of 63% for identifying limbs with clinical improvement after 6 weeks. Good angiographic result at the feet had sensitivity of 33% and specificity of 63% for identifying limbs with clinical improvement after 6 weeks.

### Sensitivity and Specificity of Perfusion Measurements for Clinical Improvement at 6 Weeks

**Figure 4.**
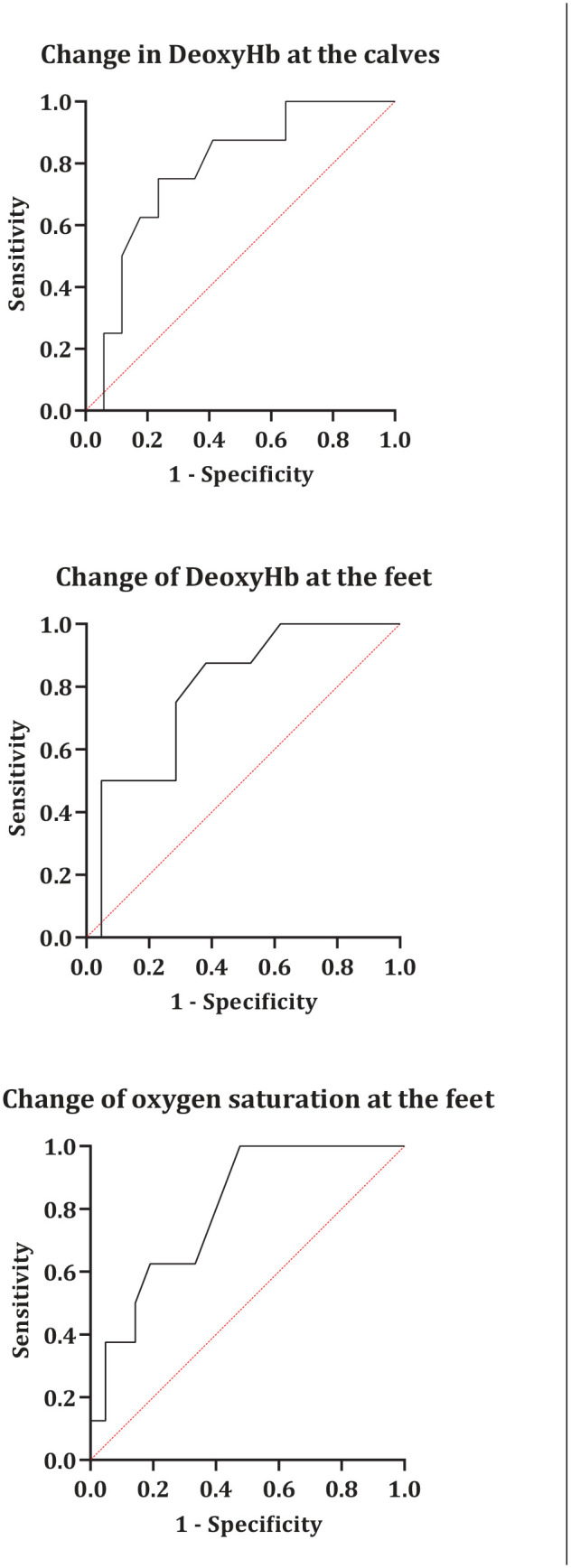
Receiver operating characteristic curves for determining the cutoff for change in deoxyhemoglobin at the calves and feet and oxygen saturation at the feet based on clinical improvement after 6 weeks. DeoxyHb, deoxyhemoglobin.

The ROC curves for changes in DeoxyHb at the calves and feet and changes in oxygen saturation at the feet are shown in Figure 4. The ROC curves were only calculated for parameters that were significantly different between limbs with and without clinical improvement.

The ROC curve showed an optimal cutoff value of ≤1.0 arbitrary units (a.u.) for change in DeoxyHb at the calves after EVT for identifying limbs with clinical improvement after 6 weeks. This optimal cutoff value for change in DeoxyHb yielded an AUC of 0.779 (95% confidence interval [CI], 0.589–0.970; p=0.027), a sensitivity of 77%, and a specificity of 75%. The ROC curve for change in DeoxyHb at the feet after EVT showed an optimal cutoff values of ≤−0.5 a.u. for identifying limbs with clinical improvement after 6 weeks. This optimal cutoff value for change in DeoxyHb at the feet yielded an AUC of 0.792 (95% CI, 0.618–0.965; p=0.017), with a sensitivity of 62% and a specificity of 88%. The ROC curve for change in oxygen saturation at the feet after EVT showed an optimal cutoff value of ≥3.0% for identifying limbs with clinical improvement after 6 weeks. This optimal cutoff value for change in oxygen saturation yielded an AUC of 0.798 (95% CI, 0.632–0.963; p=0.015), with a sensitivity of 55% and a specificity of 100%.

## Discussion

This prospective study demonstrates the ability of non-invasive HSI measurements to detect changes in lower extremity perfusion directly after endovascular interventions. A decrease of DeoxyHb at the calves and feet and an increase of oxygen saturation at the feet can be used to identify limbs that show clinical improvement 6 weeks after EVT. These changes in DeoxyHb, and an increase of oxygen saturation at the feet are significantly different from limbs without clinical improvement. The measured change in DeoxyHb has a relatively low sensitivity of 62% compared with the specificity of 88% with a cutoff of ≤−0.5. This implicates that there is a relatively high chance of missing patients that will have clinical improvement. The AUC of the ROC curve for DeoxyHb was 0.792, which means that there is an 79% change that the limbs with clinical improvement at 6 weeks can be correctly identified. An AUC of 0.7–0.8 has been considered acceptable for diagnostic accuracy.^23^ Thermal imaging was not discriminative for short-term clinical improvement.

The current finding that HSI derived DeoxyHb is the most accurate perfusion variable is in line with previous studies where DeoxyHb was the only HSI variable that was significantly different between diseased and non-diseased limbs.^11,12^ Moreover, Grambow et al, found a correlation between HSI and clinical outcomes specified as complaint-free walking distance and quality of life.^24^ Besides that, previous research found that DeoxyHb had the highest test-retest, intra-rater and inter-rater reliability of the HSI variables.^11,13^ Previous studies on HSI as a diagnostic tool failed to determine an association between HSI variables with conventional diagnostics such as ABIs.^11,12,24,25^ This is because ABI is not an adequate measure of local tissue perfusion, but merely reflects the patency of the main arterial tree of the lower extremity. On top of that, ABI measurements in patients with diabetes mellitus (DM) or Mönckeberg’s sclerosis are often falsely elevated because of incompressible vessels. The benefit of HSI may be of particularly help for this specific patient group.

This study proposes a classification based on the final angiogram as a reference standard to determine the expected effect on localized change in perfusion directly after EVT. In this way, in only 11 calves and 9 feet was the completion angiogram scored as “good” in terms of improvement of the arterial inflow of the tissue where HSI and thermal measurements were performed. Although 96% of the target lesion revascularizations were technically successful, a direct change in localized superficial tissue perfusion was not expected directly after EVT in these limbs. The ability of angiographic results to discriminate between limbs with and without clinical improvement at 6 week follow-up was low. The low sensitivity suggests that presence of uninterrupted flow in the larger arteries does not always lead to clinical improvement. A possible explanation is that the microvasculature is too much affected to regenerate. Local tissue perfusion measurements may better appreciate the microvasculature and may therefore better identify limbs that will show clinical improvement. This is in line with previous findings, in which the predictive value of successful recanalization is poor.^5^ Moreover, the change in perfusion values was not different between the limbs classified as a good angiographic result and the limbs that were not. These findings reinforce the fact that conventional diagnostics, such as DSA, are not able to accurately detect changes in local tissue perfusion and the need for a non-invasive perfusion imaging technique such as HSI seems beneficial.

There is no gold standard available for assessment of local tissue perfusion during endovascular revascularization. To date, the evaluation of treatment success is still largely based on macrovascular assessment such as angiographic results. However, an accurate assessment of local tissue perfusion is more beneficial to determine treatment efficacy, because arterial ischemic symptoms are a direct result of poorly perfused peripheral tissue.^26^ Direct assessment of tissue perfusion after revascularization procedures may guide vascular interventionalists toward patient-tailored interventions. HSI provides contact-free, non-invasive, and real-time visualization of local tissue perfusion. Other possible techniques, such as CT perfusion imaging,^27^ 2-dimensional perfusion angiography,^28^ or indocyanine green fluorescence imaging,^29^ can be performed during EVT. These techniques are, however, more invasive and more costly, and studies investigating diagnostic accuracy for clinical outcomes are lacking.

This study did not show significant changes in local skin temperature after EVT, and changes in skin temperature were not discriminative for angiographic results or clinical improvement. Previous studies reported changes in temperature after revascularization, a correlation with freedom from amputation, and a correlation with ABI.^16,17,30^ This discrepancy between our results and previous findings could be attributed to the measurement conditions. Thermal imaging in this study was performed in the angiography suite directly before and after EVT with a fixed room temperature, whereas imaging in these other studies was performed in different rooms, with higher temperatures (up to 24°C), or imaging was performed at different days. It is also likely that thermal imaging directly after EVT is too early to detect changes in skin temperature. The results of the correlation analysis showed a moderate correlation between change in temperature and change in HSI measured at the calves however not at the feet. It is possible that HSI determined at the calves is influenced by skin temperature, the reason why this is not the case for the feet remains unclear. Local hyperaemia is known to affect and influence HSI readings and can, for instance, be caused by infections. Therefore ROIs for image analysis were not placed around the wound area. Patients with DM often have a disturbed micro-vascular regulation which can lead to an altered hyperaemic response after EVT. There were no patients with active infections and there were 12 patients with DM. A sub-analysis of these DM patients to determine the correlation between temperature and HSI was not possible due to the small number of participants. Future studies on thermal imaging and HSI should further investigate this effect by including larger groups of non-diabetic and diabetic patients.

### Limitations

A limitation of the study is that a small number of limbs were included. The study population was also quite heterogeneous, including patients with Rutherford class 2–6 and patients with and without DM. Also, the patients included were classified according to the Rutherford classification; however, toe pressure and ankle pressure measurements were not available for all patients. The use of objective criteria within the Rutherford classification would have been better to distinguish between the grades of the Rutherford classification. Another limitation is the qualitative assessment of angiographic results in order to assess perfusion changes. Although such an angiographic assessment is a subjective method for determining whether perfusion is sufficiently restored, it is still a reflection of daily clinical practice during endovascular interventions. In the current study, under- or overestimation of the angiographic results could have occurred such as in patients with perfusion of the foot, without a single run off-vessel present or when patients without perfusion of the foot, had a run off-vessel present below the knee. In these patients, the angiographic classification we used may have been too coarse. The small number of limbs in the groups with and without clinical improvement in the ROC analysis resulted in a large confidence intervals. The results and predictive value of the established cutoff values for change in perfusion should therefore be verified in future studies with larger groups. Also, clinical outcome was only determined at 6 weeks of follow-up. Future studies with larger patient groups could also consider other clinical outcome measures such as wound healing, pain scores, amputation-free survival, and freedom from reintervention with lengthier follow-up.

## Conclusions

This study demonstrates the ability of HSI to detect changes in perfusion after EVT in patients with Rutherford class 2–6. Peri-procedural deoxyhemoglobin changes at the calves and feet are significantly different between limbs with and without clinical improvement at 6 week follow-up. Decrease in deoxyhemoglobin directly after EVT may identify limbs that show clinical improvement 6 weeks after EVT.

## Supplemental Material

sj-docx-1-jet-10.1177_15266028221082013 – Supplemental material for Detecting Changes in Tissue Perfusion With Hyperspectral Imaging and Thermal Imaging Following Endovascular Treatment for Peripheral Arterial Disease Click here for additional data file. Supplemental material, sj-docx-1-jet-10.1177_15266028221082013 for Detecting Changes in Tissue Perfusion With Hyperspectral Imaging and Thermal Imaging Following Endovascular Treatment for Peripheral Arterial Disease by Simone F. Kleiss, Kirsten F. Ma, Mostafa El Moumni, Çagdas Ünlü, Thomas S. Nijboer, Richte C. L. Schuurmann, Reinoud P. H. Bokkers and Jean-Paul P. M. de Vries in Journal of Endovascular Therapy

sj-docx-2-jet-10.1177_15266028221082013 – Supplemental material for Detecting Changes in Tissue Perfusion With Hyperspectral Imaging and Thermal Imaging Following Endovascular Treatment for Peripheral Arterial Disease Click here for additional data file. Supplemental material, sj-docx-2-jet-10.1177_15266028221082013 for Detecting Changes in Tissue Perfusion With Hyperspectral Imaging and Thermal Imaging Following Endovascular Treatment for Peripheral Arterial Disease by Simone F. Kleiss, Kirsten F. Ma, Mostafa El Moumni, Çagdas Ünlü, Thomas S. Nijboer, Richte C. L. Schuurmann, Reinoud P. H. Bokkers and Jean-Paul P. M. de Vries in Journal of Endovascular Therapy
