# Supplementary material for: Detecting Changes in Tissue Perfusion With Hyperspectral Imaging and Thermal Imaging Following Endovascular Treatment for Peripheral Arterial Disease
Source: J Endovasc Ther. 2022 Mar 8;30(3):382–92. doi: 10.1177/15266028221082013 (PMC10209500; doi:10.1177/15266028221082013)
Supplement: sj-docx-1-jet-10.1177_15266028221082013 – Supplemental material for Detecting Changes in Tissue Perfusion With Hyperspectral Imaging and Thermal Imaging Following Endovascular Treatment for Peripheral Arterial Disease [file sj-docx-1-jet-10.1177_15266028221082013.docx]

| **Supplementary Table 1.** HSI values and skin temperature of the calves and feet before and after EVT in limbs classified as good angiographic result. | | | | |
| --- | --- | --- | --- | --- |
|  |  | Pre-EVT | Post-EVT | p value |
| Calves | N= 10 |  |  |  |
| HSI measurements | Oxyhemoglobin (a.u.) | 24.0 (15.8, 31.3) | 30.0 (23.8, 52.3) | **0.050** |
|  | Deoxyhemoglobin (a.u.) | 42.0 (32.8, 47.5) | 35.0 (28.0, 44.5) | 0.528 |
|  | Oxygen saturation (%) | 40.5 (24.0, 48.3) | 54.5 (35.5, 64.0) | **0.050** |
|  | N=11 |  |  |  |
| Thermal imaging | Temperature (°C) | 33.0 (32.3, 34.2) | 33.5 (32.0, 35.1) | 0.838 |
|  |  |  |  |  |
| Feet | N=9 |  |  |  |
| HSI measurements | Oxyhemoglobin (a.u.) | 78.0 (71.5, 97.5) | 93.0 (72.5, 102.0) | 0.285 |
|  | Deoxyhemoglobin (a.u.) | 46.0 (40.5, 80.5) | 59.0 (40.0, 82.5) | 0.673 |
|  | Oxygen saturation (%) | 63.0 (52.5, 65.0) | 61.0 (51.0, 68.0) | 0.677 |
| Thermal imaging | Temperature (°C) | 30.3 (27.0, 33.4) | 32.8 (31.0, 34.0) | 0.139 |
| Values are presented as median and interquartile range. Wilcoxon signed rank test was performed to calculate differences between variables before and after EVT. HSI= hyperspectral imaging. EVT= endovascular treatment. a.u.: arbitrary units. Bold *p* values are statistically significant (p ≤.05). Included numbers were lower at the calves because of missing images. | | | | |
