# Supplementary material for: Detecting Changes in Tissue Perfusion With Hyperspectral Imaging and Thermal Imaging Following Endovascular Treatment for Peripheral Arterial Disease
Source: J Endovasc Ther. 2022 Mar 8;30(3):382–92. doi: 10.1177/15266028221082013 (PMC10209500; doi:10.1177/15266028221082013)
Supplement: sj-docx-2-jet-10.1177_15266028221082013 – Supplemental material for Detecting Changes in Tissue Perfusion With Hyperspectral Imaging and Thermal Imaging Following Endovascular Treatment for Peripheral Arterial Disease [file sj-docx-2-jet-10.1177_15266028221082013.docx]

| **Supplementary Table 2.** HSI values and skin temperature of the calves and feet before and after EVT in the 21 limbs that showed clinical improvement 6 weeks post-EVT | | | | |
| --- | --- | --- | --- | --- |
|  |  | Pre-EVT | Post-EVT | p value |
| Calves | N=17 |  |  |  |
| HSI measurements | Oxyhemoglobin (a.u.) | 22.0 (15.5, 29.0) | 27.5 (18.8, 43.5) | 0.244 |
|  | Deoxyhemoglobin (a.u.) | 44.0 (40.5, 58.0) | 37.0 (28.0, 57.3) | **0.010** |
|  | O2 saturation (%) | 30.0 (20.5, 43.5) | 41.0 (26.5, 54.5) | 0.057 |
|  | N=18 |  |  |  |
| Thermal imaging | Temperature (°C) | 32.6 (32.2, 33.9) | 33.3 (32.1, 35.1) | 0.193 |
|  |  |  |  |  |
| Feet | N= 21 |  |  |  |
| HSI measurements | Oxyhemoglobin (a.u.) | 78.0 (52.0, 97.0) | 81.0 (58.0, 100.0) | 0.487 |
|  | Deoxyhemoglobin (a.u) | 61.0 (54.5, 84.5) | 60.0 (48.0, 75.5) | 0.056 |
|  | Oxygen saturation (%) | 56.0 (43.0, 61.0) | 55.0 (47.0, 65.5) | 0.056 |
| Thermal imaging | Temperature (°C) | 30.1 (24.9, 32.6) | 32.1 (25.2, 34.1) | 0.170 |
| Values are presented as median and interquartile range. Differences between perfusion values pre and post EVT were determined with a Wilcoxon rank test. HSI= hyperspectral imaging. EVT= endovascular treatment. a.u.: arbitrary units. Bold p values are statistically significant (p ≤ .05). Included numbers were lower at the calves because of missing images. | | | | |
